# Supplementary material for: Patterns of Intron Gain and Loss in Fungi
Source: PLoS Biol. 2004 Nov 30;2(12):e422. doi: 10.1371/journal.pbio.0020422 (PMC532390; doi:10.1371/journal.pbio.0020422)
Supplement: Table S1 — Also available at http://genes.mit.edu/NielsenEtAl/. (4.3 MB ZIP). [file pbio.0020422.st001.zip › NielsenEtAl/html/612.html]

AN9083.1.NCU02090.1.MG00657.1.FG00722.1


```
 CLUSTAL W (1.82) Multiple Sequence Alignments - Introns Inserted


Sequence 1: MG00657.1	216 aa
Sequence 2: FG00722.1	215 aa
Sequence 3: NCU02090.1	226 aa
Sequence 4: AN9083.1	214 aa
Alignment Length: 233 aa
Number Identitical Residues: 95 aa
Alignment Score (without introns) 4630


MG00657.1 	--------------MSSTDPTTTGQT---AQDASRQQPTAAASSSSELASAKITLYKVLR
NCU02090.1	MSANAESAPQAADLVTSTQDNTANTQSTISQDAAGLESSSSPAAAAELASTKVKLMAALR
FG00722.1 	MSA--------QDPLSSTNPSSVTNN---TQDASGRQPSSAAAGSSELSSAKISLRKSLR
AN9083.1  	----------MSQHTASTSPDASSTT-----QVAG-HPSSATSRAAELASLKVKLRGALR
          	            .  :**.  :         :.:  ..:::.: ::**:* *:.*   **

MG00657.1 	RFADFPIEGIVFIDIMPIFIDPKAHLALLRALELQVLSSFAPATAKPDVVVGLDARGFLF
NCU02090.1	SFPDFPIPGINFIDILPLFQDVKTHEALLVALQLQIELSHQ----KPDVIVGLDARGFLF
FG00722.1 	SFPDFPIPGINFVDIMPLFADPTAHATLVEALELQIAEAFP---TKPDVIVGLDARGFLF
AN9083.1  	QFPDFPSPGILFEDILPIFSDHSTHEALVRCLELHILETLAD--EKPDVIVGLEARGFLM
          	 *.***  ** * **:*:* * .:* :*: .*:*::  :      ****:***:*****:

MG00657.1 	GPSLALKLDASFVPVRKKGKLPGPCVTATYEKEYGQDFFQMQEGAIKPGQKVLVVDDIIA
NCU02090.1	GPSLALRMGASFVPVRKQGKMPGPCVTAAYEKEYGTDFFQMQDGAIKPGQKVLIVDDIIA
FG00722.1 	GPGLALRLGVPFAAVRKQGKLPGPCVTAEYIKEYGKDLFQMQEDAVREGQKVLIVDDIIA
AN9083.1  	GPSLALKLGAGFVPVRKRGKLPGPCETQGYEKEYGQDFFQMQSDAIKPGQKVIVIDDIIA
          	**.***::.. *..***:**:**** *  * **** *:****..*:: ****:::*****

MG00657.1 	TG1GSAAAAGELVKKLGGELMGYLFLLEIAPLKGREKLGGVPVVTLLEEADYPH
NCU02090.1	TG1GSAAAAGELVKKLGGETIGYLFILEIAFLKGREKLGGVPVTTLLETDE---
FG00722.1 	TG1GSAKAAAELVQQLKGEVMGYLFILEIPGLNGRDKLGAPPTKILLEDA----
AN9083.1  	TG1GSAYAAGELIKKMGGNVLGFVFLLELEFLHGRDKLS-APVHTLLTGQETKA
          	** *** **.**:::: *: :*::*:**:  *:**:**.  *.  **   .
```
